# The ABCA1 agonist CS6253 shows favorable safety and pharmacokinetics in IND‐enabling toxicology and Phase 1 human studies aimed at the indication of Alzheimer’s disease

**DOI:** 10.1002/alz.091450

**Published:** 2025-01-09

**Authors:** Jan O. Johansson, Bengt Winblad, Daniel M Michaelson, Henrik Zetterberg, Jeffrey L. Cummings, Hussein N Yassine

**Affiliations:** ^1^ Artery Therapeutics, Inc., San Ramon, CA USA; ^2^ Karolinska Institutet, Stockholm Sweden; ^3^ Tel Aviv University, Tel Aviv Israel; ^4^ Hong Kong Center for Neurodegenerative Diseases, Hong Kong China; ^5^ University of Gothenburg, Mölndal, Gothenburg Sweden; ^6^ UCL Institute of Neurology, Queen Square, London United Kingdom; ^7^ Department of Psychiatry and Neurochemistry, Institute of Neuroscience and Physiology, The Sahlgrenska Academy at the University of Gothenburg, Mölndal Sweden; ^8^ Chambers‐Grundy Center for Transformative Neuroscience, University of Nevada, Las Vegas, NV USA; ^9^ Keck School of Medicine at University of Southern California, Los Angeles, CA USA

## Abstract

**Background:**

ABCA1‐mediated cholesterol transport is a central feature in many lipid‐ dependent diseases including APOE4‐associated Alzheimer’s disease and atherosclerosis‐CVD. ABCA1 upregulation of RNA transcription by nuclear factors (LXR, RXR) have been associated with liver side‐effects because of the common promotor element for ABCA1 and Fatty Acid Synthase. The ABCA1 agonist CS6253, derived from the C‐terminal of apoE was designed to stabilize and enhance ABCA1 function, thereby providing a safe alternative to transcriptional upregulation. CS6253 has in various mice models shown favorable neuroprotective and vascular‐metabolic effects suggesting potential for APOE4 MCI/AD and mixed MCI/AD.

IND enabling studies in Wistar rats and Cynomolgus monkeys(cynos) were performed, and a Phase 1 SAD‐MAD study initiated, to explore the safety, PK and biomarker effects of CS6253.

**Method:**

Conventional IND enabling toxicology studies, including 30‐day GLP studies in male and female rats and cynos were performed, in which CS6253 was injected as IV bolus injection every other day. Following clearing the IND a Phase 1 SAD‐MAD double‐blind, placebo‐controlled study was initiated, including elderly with and without APOE4 genotype, the SAD part ending in March 2024.

**Result:**

Toxicology studies showed that cynos was the more sensitive species with the No Observable Adverse Effect Level (NOAEL) being 75 mg/kg. In plasma exposure/AUC in cynos was linear up to 25 mg/kg and showed already from 10 mg/kg transient increases in preb1‐HDL, small HDL and triglycerides which was associated with a significant increase in amyloidb42/40‐ratio.

**Conclusion:**

Enhancing ABCA1 functions by CS6253 to form small HDL appears to be a promising approach with neuroprotective properties as indicated by pharmacology, IND‐enabling toxicology, and Phase 1 studies. The development of a safe and efficient ABCA1 agonist addresses several indications with unmet medical, notably for Alzheimer’s and brain vascular diseases.

